# Mapping health-related quality of life scores from FACT-G, FAACT, and FACIT-F onto preference-based EQ-5D-5L utilities in non-small cell lung cancer cachexia

**DOI:** 10.1007/s10198-017-0930-6

**Published:** 2017-09-25

**Authors:** Michela Meregaglia, Ludovica Borsoi, John Cairns, Rosanna Tarricone

**Affiliations:** 1 CeRGAS (Research Centre on Health and Social Care Management), Bocconi University, Via Roentgen 1, 20136 Milan, Italy; 2 Department of Health Services Research, London School of Hygiene and Tropical Medicine (LSHTM), London, UK; 3 Department of Policy Analysis and Public Management, Bocconi University, Milan, Italy; 4 CCBIO (Centre for Cancer Biomarkers), University of Bergen, Bergen, Norway

**Keywords:** Mapping, FAACT, FACIT-F, FACT-G, EQ-5D-5L, Non-small cell lung cancer cachexia, C2, I1

## Abstract

**Background:**

Health-related quality of life (HRQoL) measurements from disease-specific tools cannot be directly used in economic evaluations. This study aimed to develop and validate mapping algorithms that predicted EuroQol 5-Dimensions 5-Levels (EQ-5D-5L) utilities from Functional Assessment of Anorexia-Cachexia Therapy (FAACT) and Functional Assessment of Chronic Illness Therapy-Fatigue (FACIT-F) and their common component (Functional Assessment of Cancer Therapy-General—FACT-G) in patients with non-small cell lung cancer cachexia.

**Methods:**

Data were collected on five occasions over a 12-week period in two multicenter placebo-controlled trials. EQ-5D-5L utilities were calculated using both English and Dutch value sets. The study sample was divided into development and validation datasets according to patients’ geographical residence. Generalized estimating equations were applied to five different sets of independent variables including overall, Trial Outcome Index (TOI), and individual subscales results. The best performing models were selected based on mean absolute error (MAE) and root-mean square error (RMSE).

**Results:**

EQ-5D-5L and FAACT/FACIT-F results were available for 96 patients. The developed algorithms showed a good predictive performance, with acceptable MAE/RMSE and small differences between mean observed and predicted EQ-5D-5L utilities. In FACT-G models, Physical Well-Being had the highest explanatory value, while Emotional Well-Being did not significantly affect the EQ-5D-5L score; Anorexia-Cachexia and Fatigue subscales were highly statistically significant in FAACT and FACIT-F models, respectively, as well as the TOI scores. The Eastern Cooperative Oncology Group status was included as covariate in all models.

**Conclusion:**

The developed algorithms enable the estimation of EQ-5D-5L utilities from three cancer-specific instruments when preference-based HRQoL data are missing.

**Electronic supplementary material:**

The online version of this article (doi:10.1007/s10198-017-0930-6) contains supplementary material, which is available to authorized users.

## Introduction

Cachexia has been defined as “a complex metabolic syndrome associated with underlying illness and characterized by the loss of muscle with or without the loss of fat mass” [1]. Cachexia is a common clinical manifestation in cancer, especially at advanced stages. The frequency of this syndrome in lung cancer consistently outweighs that in other malignancies [2]. Cachectic patients experience a wide range of symptoms including lack of appetite, early satiety, and impaired physical functioning, which cumulatively decrease their quality of life and worsen their prognosis. Indeed, cachexia is estimated to be the direct cause of at least 20% of cancer deaths [3, 4].

The questionnaires belonging to the functional assessment of chronic illness therapy (FACIT) measurement system [5] are frequently adopted to estimate health-related quality of life (HRQoL) in cancer studies. The functional assessment of cancer therapy-general (FACT-G) can be used in any tumor type and constitutes the core of all other questionnaires addressing the specific concerns of patients with various malignancies. Among them, the functional assessment of anorexia/cachexia treatment (FAACT) has been recommended in the assessment of cancer-related cachexia [6]. The questionnaire may be administered in conjunction with others such as the functional assessment of chronic illness therapy-fatigue (FACIT-F), which is specifically aimed at measuring fatigue symptoms in chronic diseases. However, these questionnaires do not provide preference-based scores (utilities) that are essential in the quality-adjusted life years (QALYs) calculation for cost-effectiveness analyses. Conversely, the EuroQol five-dimensional (EQ-5D) questionnaire, developed by the group EuroQol [7], furnishes preference weights from the general population to derive QALYs and has been endorsed as a health utility standard by the National Institute for Health and Care Excellence (NICE) in England [8].

The measurement of HRQoL in oncology is mostly carried out using cancer-specific instruments rather than generic preference-based measures as they focus on relevant health issues and tend to capture more meaningful changes in symptomatology [9]. In a systematic literature review of studies addressing HRQoL in cancer anorexia-cachexia syndrome [10], FAACT was endorsed by seven studies, one of them reporting also the common core component (FACT-G), while only four studies adopted the generic EQ-5D instrument. In the absence of preference-based scores, statistical models that ”map”, or “cross-walk”, the responses from a disease-specific instrument to utility values can be used as an alternative solution. In recent years, there has been a growing interest in mapping, with a number of published algorithms predicting EQ-5D health utilities from a wide range of disease-specific, non-preference-based scores [11]. A database maintained by the Health Economics Research Centre (Oxford University) [12] and regularly updated with all mapping studies published in the literature yielded 24 algorithms estimating EQ-5D scores from cancer-specific instruments; among them, eight studies adopted a questionnaire from the FACIT group to address a variety of tumors (breast, prostate, colorectal, lung, and melanoma). However, no algorithm exists specifically for anorexia-cachexia cancer syndrome, thus making it difficult for regulatory bodies to assess the QALY gain of new treatments for the purpose of reimbursement in the absence of generic preference-based data collections. The objective of this study was to develop a reliable mapping function to estimate the 5-level EQ-5D (EQ-5D-5L) utility values from FACT-G, FAACT, and FACIT-F scores to inform future cost-effectiveness analyses in the cancer cachexia setting or in lung cancer.

## Methods

In performing this study, we referred to the recently published MAPS reporting statement [13] and completed the proposed 23-item checklist for mapping studies (Table A1, online appendix).

### Study sample

Data from two multicenter, randomized, double blind, placebo-controlled phase 3 trials (ROMANA 1 and ROMANA 2) in patients with non-small cell lung cancer-cachexia (NSCLC-C) were used for this analysis. The trials were conducted at 93 sites in 19 countries between 2011 and 2014. The two studies enrolled a total of 979 patients aged ≥18 years with a diagnosis of stage III or IV NSCLC and cachexia [defined as involuntary loss of ≥5% body weight within 6 months or body mass index (BMI) <20 kg/m^2^]; patients had an estimated life expectancy of more than 4 months at enrollment and a Eastern Cooperative Oncology Group (ECOG) performance status ≤2. Details of the inclusion/exclusion criteria and the study design are described elsewhere [14]. Patients were randomly assigned 2:1 to receive active treatment (100 mg, Anamorelin HC1, Helsinn Therapeutics, Inc.) or placebo once daily over a 12-week period. Primary efficacy endpoints were the median change in lean body mass and handgrip strength over the same period. Among the secondary efficacy parameters, HRQoL was assessed by FACIT-F and FAACT (version 4) at baseline and weeks 3, 6, 9, and 12. Moreover, the EQ-5D-5L questionnaire was administered at the same time points to a subset of patients in only two sites (i.e. Poland and Hungary). For the purpose of this analysis, we used the sample of observations reporting both HRQoL instruments (i.e. FACIT-F/FAACT and EQ-5D-5L) without any distinction between the treatment arms.

### Instruments

The EQ-5D-5L questionnaire is a generic, preference-based HRQoL measure comprising five domains: mobility, self-care, usual activities, pain/discomfort, and anxiety/depression [7]. Each dimension has five levels: no problems, slight problems, moderate problems, severe problems, and extreme problems. Patients’ responses to the questionnaire were scored using the English EQ-5D-5L value set [15], which ranges from −0.281 (state 55555) and 1 (state 11111, representing perfect health) and the Dutch EQ-5D-5L value set [16] ranging between −0.446 and 1 to illustrate the sensitivity of results to the use of alternative value sets.

The FAACT is the anorexia-cachexia-specific HRQoL instrument of the FACIT system [5]. It comprises the 27-item FACT-G and a 12-item Anorexia-Cachexia Subscale (ACS). Each item is rated on a 5-point Likert scale (from 0 to 4) ranging from “not at all” to “very much”. The FAACT is the sum of the FACT-G score (0–108) and the ACS score (0–48) with higher values representing better health. Similar to FAACT, the FACIT-F comprises the FACT-G and a 13-item Fatigue Subscale (0–52), yielding a total score between 0 and 160. The FACT-G is composed of four subscales assessing physical wellbeing (PWB, 0–28), functional wellbeing (FWB, 0–28), social/family well-being (SWB, 0–28), and emotional wellbeing (EWB, 0–24). From these scales, it is also possible to calculate a Trial Outcome Index (TOI), which is the sum of PWB, FWB, and tool-specific subscales (in this study, ACS and Fatigue).

### Statistical analysis

Generalized estimating equations (GEEs) were performed in order to derive mapping functions for FAACT, FACIT-F, and FACT-G. GEE is a technique facilitating the analysis of data collected in longitudinal, clustered, or repeated measures designs, which is increasingly applied in clinical trials and biomedical studies. GEE is a population-level approach based on a quasi-likelihood function that provides the population-averaged estimates of the parameters. GEEs use the generalized linear model to estimate regression parameters allowing the specification of a working correlation matrix that accounts for the type of within-subject correlation of responses on the dependent variable [17, 18]. The GEE method was selected because of the longitudinal nature of the ROMANA trials, where repeated observations from each instrument are expected to be correlated between visits. This method has been used previously to predict EQ-5D utilities from the Functional Assessment of Cancer Therapy-Prostate (FACT-P) using data from a multicenter, randomized, placebo-controlled trial [19]. Quasi-likelihood under the independence model criterion (QIC) statistics were calculated in order to select the best-working correlation structure [20].

In order to identify the best model specification, five alternative sets of explanatory variables were compared. Models were developed in order of increasing complexity given by the level of disaggregation of FACIT questionnaires scores. In detail, EQ-5D-5L scores were predicted from the overall FACT-G, FAACT, and FACIT-F scores (Model 1), from the generic (FACT-G) and specific (ACS and Fatigue) components separately (Model 2), from the four domains that compose FACT-G (PWB, FWB, SWB, and EWB) (Model 3), from the TOI score alone (Model 4) and from the three components (PWB, FWB, and ACS or Fatigue) of TOI scores (Model 5). Selected clinical and demographic variables were tested for potential inclusion in the final models on the basis of their statistical significance. These variables were: age (>65 or ≤65), gender, body mass index (BMI; ≤18.5 or >18.5 kg/m^2^), ECOG (0–1 or 2) performance at baseline, weight loss in previous 6 months (> or ≤10% of body weight) and current chemotherapy/radiotherapy status (yes or no). The great majority (99.0%) of the patients enrolled were white, thus ethnicity was not considered as a covariate in the regression models [21]. Squared FACIT scores were tested as well to allow for nonlinear relationships with EQ-5D-5L utility. No imputation of missing values was performed in order to avoid assumptions about regarding early patient dropout. No interactions were tested, as previous research demonstrated that adding interaction terms seldom improved the model fit [11, 21, 22] and the “principle of parsimony” should be embraced in developing mapping algorithms so that they can be more readily used by future researchers [23]. Pearson’s correlation was performed to estimate the degree of conceptual overlap between the source(s) and target measures that justified a mapping exercise among them (Table A2, online appendix).

### Model selection and cross-validation

The performance of each model was assessed in terms of how well the responses to FACT-G, FAACT, and FACIT-F predicted EQ-5D-5L utilities. An out-of-sample validation is usually recommended to test the algorithms; however, no external datasets were available and an internal cross-validation technique was applied to derive goodness-of-fit statistics. The study sample was non-randomly divided into two groups using one-fifth (validation sample) four-fifths (development sample) split according to patients’ geographical residence (i.e. Poland or Hungary). Following the approach of a previous study [24] and recommendations from the MAPS statement [13], we assumed that a non-random split ensures a more efficient validation, as the two groups are likely to differ according to some baseline characteristics. Statistical tests (i.e. Chi-squared for categorical variables and *t* test for continuous variables) were performed in order to explore the differences between the two sub-samples. Mapping functions were fitted on the development sample, while the remaining observations were used to test the models’ performance.

Model validation was performed by pooling all the visits together in order to obtain average performance indexes within the database. The mean absolute error (MAE) and root-mean square error (RMSE) were calculated to examine the differences between mean observed and predicted EQ-5D-5L scores, with lower values indicating better algorithm performance. The MAE is the average of absolute differences between observed and predicted utilities, while the RMSE is the root of the average of the squared differences. A paired *t* test (*p* < 0.05) was also applied to the differences between observed and mapped EQ-5D-5L scores with significant results indicating low predictive accuracy [25]. The best performing models were selected on the basis of the lowest MAE/RMSE results. Moreover, these differences were compared to the minimal important difference (MID) in EQ-5D utility that, in cancer patients, has been estimated as 0.08 using the UK value set for the 3-level version (EQ-5D-3L) [26].

All analyses were conducted using STATA version 14.1 (College Station, TX, USA) and Microsoft Excel 2013.

## Results

### Descriptive statistics

Demographic and baseline clinical data stratified by overall, development, and validation dataset are reported in Table 1. Overall, 96 patients completed both EQ-5D-5L and FACIT questionnaires; the number of observations per patient ranged between 1 and 5, for a total of 420 of which 332 were used to develop the algorithms and the remaining 88 for validating them. In the overall sample (*n* = 96), the majority of patients were male (68.7%) and their mean age was 61 years; most (86.5%) were on chemotherapy or radiotherapy treatments. The average EQ-5D-5L utility at baseline was 0.766 (SD = 0.19), ranging between −0.102 and 1 (perfect health); mean FACT-G, FAAC,T and FACIT-F overall scores were 64.9 (SD = 14.2), 93.2 (SD = 21.0), and 93.2 (SD = 22.4), respectively. Among FACT-G subscales, SWB had the highest score (20.5 ± 4.7), whilst EWB had the lowest (13.4 ± 5.0).

**Table 1:** Sample characteristics and HRQoL scores (at baseline) stratified by study dataset

Variable	Statistics	Total sample (*n* = 96)	Development sample (*n* = 76)	Validation sample (*n* = 20)	*p* value
Demographic and clinical data					
Country	Poland [*N* (%)] Hungary [*N* (%)]	76 (79.2) 20 (20.8)	76 (100.0) 0 (0.0)	0 (0.0) 20 (100.0)	
Age (years)	Mean (±SD) Range (min, max)	61.1 (±8.7) 36, 85	61.3 (±9.0) 36, 85	60.5 (±7.7) 46, 80	0.7217^a^
Gender	Male [*N* (%)]	66 (68.7)	54 (71.0)	12 (60.0)	0.343^b^
BMI (Kg/m^2^)	≤18.5 [*N* (%)]	7 (7.29)	3 (3.95)	4 (20.0)	0.014^b*^
Weight loss (in previous 6 months)	>10% of body weight [*N* (%)]	39 (40.6)	25 (32.9)	14 (70.0)	0.003^b,**^
ECOG	0–1 [*N* (%)] 2 [*N* (%)]	78 (81.2) 18 (18.8)	58 (76.3) 18 (23.7)	20 (100.0) 0 (0.0)	0.016^b,*^
On chemotherapy/radiotherapy	Yes [*N* (%)]	83 (86.5)	74 (97.4)	9 (45.0)	0.000^b,***^
HRQoL scores (at baseline)					
FAACT	Mean (± SD) Range (min, max)	93.2 (±21.0) 28.0, 141.0	93.4 (±21.8) 28.0, 141.0	92.6 (±17.8) 47.8, 129.0	0.8774^a^
FACIT-F	Mean (±SD) Range (min, max)	93.2 (±22.4) 20.0, 140.0	92.6 (±23.2) 20.0, 140.0	95.4 (±19.6) 57.8, 134.0	0.6274^a^
FACT-G	Mean (±SD) Range (min, max)	64.9 (±14.2) 18.0, 95.0	65.4 (±14.7) 18.0, 95.0	63.0 (±12.0) 36.8, 88.0	0.5086^a^
PWB	Mean (±SD) Range (min, max)	16.2 (±5.1) 4.0, 28.0	15.7 (±5.2) 4.0, 28.0	17.8 (±4.3) 11.0, 27.0	0.0976^a^
FWB	Mean (±SD) Range (min, max)	14.8 (±5.3) 1.0, 26.0	15.8 (±4.9) 3.0, 26.0	11.0 (±5.4) 1.0, 21.0	0.0003^a,**^
SWB	Mean (±SD) Range (min, max)	20.5 (±4.7) 4.0, 28.0	20.6 (±5.0) 4.0, 28.0	19.8 (±3.5) 12.8, 26.0	0.4864^a^
EWB	Mean (±SD) Range (min, max)	13.4 (±5.0) 0.0, 24.0	13.2 (±5.4) 0.0, 24.0	14.3 (±3.5) 7.0, 21.0	0.3800^a^
FAACT (ACS)	Mean (±SD) Range (min, max)	28.3 (±9.0) 5.0, 46.0	28.0 (±9.4) 5.0, 46.0	29.5 (±7.4) 11.0, 43.0	0.4957^a^
FACIT-F (fatigue)	Mean (±SD) Range (min, max)	28.3 (±9.8) 2.0, 49.0	27.2 (±9.9) 2.0, 49.0	32.3 (±8.2) 17.0, 46.0	0.0360^a,*^
FAACT (TOI)	Mean (±SD) Range (min, max)	59.3 (±15.9) 23.0, 99.0	59.5 (±16.3) 23.0, 99.0	58.4 (±14.5) 26.0, 85.0	0.7840^a^
FACIT-F (TOI)	Mean (±SD) Range (min, max)	59.3 (±16.9) 15.0, 98.0	58.8 (±17.2) 15.0, 98.0	61.2 (±16.1) 36.0, 90.0	0.5643^a^
EQ-5D-5L (UK index)	Mean (±SD) Range (min, max)	0.766 (±0.19) −0.102, 1.000	0.746 (±0.20) −0.102, 1.000	0.841 (±0.13) 0.527, 1.000	0.0478^a,*^
EQ-5D-5L (NL index)	Mean (±SD) Range (min, max)	0.702 (±0.23) −0.289, 1.000	0.678 (±0.25) −0.289, 1.000	0.792 (±0.15) 0.383, 1.000	0.0514^a,*^

*SD* standard deviation; *BMI* body mass index, *ECOG* Eastern Cooperative Oncology Group, *FACT-G* functional assessment of cancer therapy-general, *FAACT* functional assessment of anorexia/cachexia treatment, *FACIT-F* functional assessment of chronic illness therapy-fatigue, *PWB* physical wellbeing, *FWB* functional wellbeing, *EWB* emotional wellbeing, *SWB* social/family wellbeing, ACS Anorexia-Cachexia Subscale, *TOI* trial outcome index, *EQ-5D-5L* EuroQol five-dimension five-level, *UK* United Kingdom, *NL* Netherlands

*p value <0.05

**p value <0.01

***p value <0.001

^a^*T* test

^b^Chi-square test

**Fig. 1:**
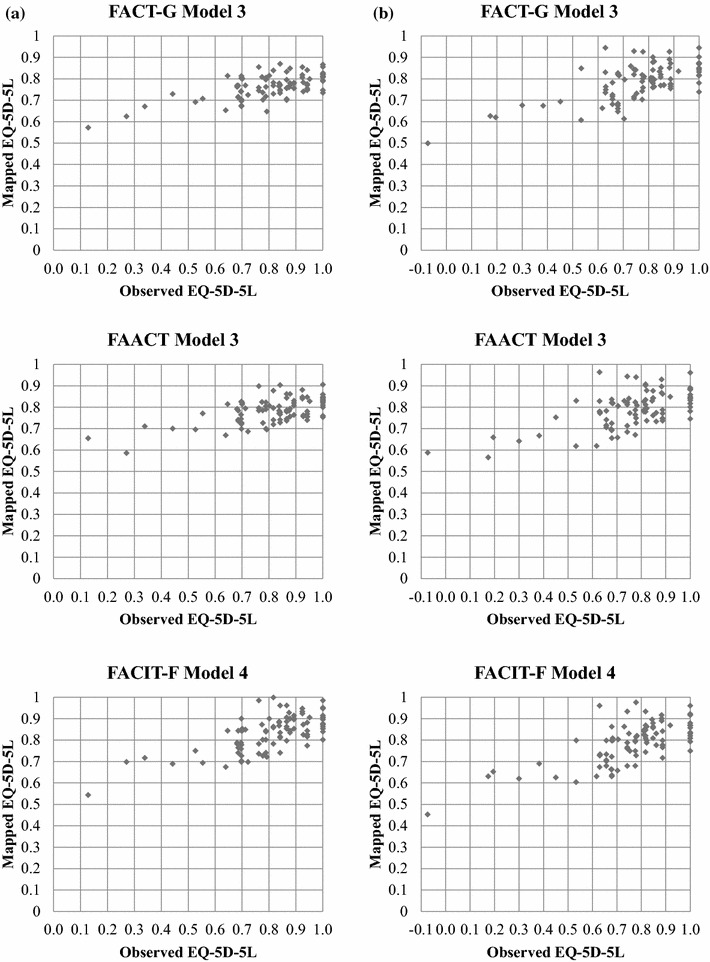
**a** Scatterplot of observed versus predicted EQ-5D-5L utility across all visits using the best performing models (validation sample, UK weights). **b** Scatterplot of observed versus predicted EQ-5D-5L utility across all visits using the best performing models (validation sample, NL weights)

Patients in the development (*n* = 76, Poland) and validation (*n* = 20, Hungary) samples differed by BMI (kg/m^2^), weight loss, ECOG performance score and chemotherapy/radiotherapy status at baseline. Significant differences (*p* < 0.05) in baseline HRQoL scores were only observed in mean EQ-5D-5L (both for English and Dutch values), FWB and Fatigue scores. Full descriptive statistics of EQ-5D-5L, FAACT and FACIT-F scores by visit for the overall sample are shown in Table 2. The distribution of EQ-5D-5L scores is shown in Fig. 1.

**Table 2:** Descriptive statistics of HRQoL observed scores by study visits (overall sample)

Instrument	Statistics	Baseline	Week 3	Week 6	Week 9	Week 12
FAACT (overall) Theoretical range (min, max): 0, 156	*N*	96	89	85	78	76
	Mean (±SD)	93.2 (±21.0)	100.5 (±22.6)	101.5 (±21.2)	100.3 (±20.7)	95.3 (±23.6)
	Range (min, max)	28.0, 141.0	57.0, 148.0	41.0, 147.7	55.3, 152.0	45.5–156.0
	% floor, % ceiling	0.0, 0.0	0.0, 0.0	0.0, 0.0	0.0, 0.0	0.0, 1.3
FACIT-F (overall) Theoretical range (min, max): 0, 160	*N*	96	89	85	78	75
	Mean (±SD)	93.2 (±22.4)	99.2 (±24.4)	99.1 (±23.0)	97.1 (±21.9)	92.0 (±26.2)
	Range (min, max)	20.0, 140.0	34.3, 154.0	37.0, 146.0	51.3, 156.0	13.5, 160.0
	% floor, % ceiling	0.0, 0.0	0.0, 0.0	0.0, 0.0	0.0, 0.0	0.0, 1.3
FACT-G Theoretical range (min, max): 0, 108	*N*	96	89	85	78	76
	Mean (±SD)	64.9 (±14.2)	69.1 (±15.4)	69.3 (±14.1)	68.2 (±13.7)	64.6 (±16.3)
	Range (min, max)	18.0, 95.0	31.3, 102.0	33.0, 101.7	41.0, 104.0	12.5, 108.0
	% floor, % ceiling	0.0, 0.0	0.0, 0.0	0.0, 0.0	0.0, 0.0	0.0, 1.3
PWB Theoretical range (min, max): 0, 28	*N*	96	90	86	78	76
	Mean (±SD)	16.2 (±5.1)	17.1 (±5.6)	17.5 (±5.8)	17.1 (±5.3)	16.3 (±5.9)
	Range (min, max)	4.0, 28.0	1.0, 28.0	1.0, 28.0	3.0, 28.0	0.0, 28.0
	% floor, % ceiling	0.0, 1.0	0.0, 2.2	0.0, 2.3	0.0, 3.8	1.3, 3.9
FWB Theoretical range (min, max): 0, 28	*N*	96	90	85	78	76
	Mean (±SD)	14.8 (±5.3)	15.8 (±5.5)	15.7 (±5.4)	15.5 (±4.7)	14.7 (±5.4)
	Range (min, max)	1.0, 26.0	0.0, 27.0	3.0, 28.0	4.0, 27.0	2.0, 28.0
	% floor, % ceiling	0.0, 0.0	1.1, 0.0	0.0, 1.2	0.0, 0.0	0.0, 1.3
SWB Theoretical range (min, max): 0, 28	*N*	96	90	86	78	76
	Mean (±SD)	20.5 (±4.7)	21.1 (±4.4)	20.9 (±4.2)	20.6 (±4.4)	20.4 (±4.2)
	Range (min, max)	4.0, 28.0	9.3, 28.0	8.0, 28.0	9.0, 28.0	10.5, 28.0
	% floor, % ceiling	0.0, 5.2	0.0, 8.9	0.0, 5.8	0.0, 5.1	0.0, 10.5
EWB Theoretical range (min, max): 0, 24	*N*	96	89	85	78	76
	Mean (±SD)	13.4 (±5.0)	14.9 (±4.7)	15.1 (±4.5)	14.9 (±4.3)	13.3 (±5.4)
	Range (min, max)	0, 24	4, 24	1, 23	6, 24	0, 24
	% floor, % ceiling	2.1, 2.1	0.0, 1.1	0.0, 0.0	0.0, 1.3	1.3, 2.6
FAACT (ACS) Theoretical range (min, max): 0, 48	*N*	96	90	86	78	76
	Mean (±SD)	28.3 (±9.0)	31.4 (±9.3)	32.1 (±8.4)	32.2 (±8.9)	30.7 (±9.3)
	Range (min, max)	5.0, 46.0	8.0, 47.0	8.0, 47.0	10.0, 48.0	8.0, 48.0
	% floor, % ceiling	0.0, 0.0	0.0, 0.0	0.0, 0.0	0.0, 1.3	0.0, 2.6
FACIT-F (Fatigue) Theoretical range (min, max): 0, 52	*N*	96	90	86	78	75
	Mean (±SD)	28.3 (±9.8)	30.2 (±10.8)	29.8 (±10.4)	29.0 (±10.1)	27.8 (±10.9)
	Range (min, max)	2.0, 49.0	3.0, 52.0	1.0, 50.0	6.0, 52.0	1.0, 52.0
	% floor, % ceiling	0.0, 0.0	0.0, 1.1	0.0, 0.0	0.0, 2.6	0.0, 1.3
FAACT (TOI) Theoretical range (min, max): 0, 104	*N*	96	89	85	78	76
	Mean (±SD)	59.32 (±15.88)	64.51 (±17.16)	65.44 (±16.71)	64.79 (±15.68)	61.60 (±17.46)
	Range (min, max)	23, 99	24, 98	12, 100	21, 102	28, 104
	% floor, % ceiling	0.0, 0.0	0.0, 0.0	0.0, 0.0	0.0, 0.0	0.0, 1.3
FACIT-F (TOI) Theoretical range (min, max): 0, 108	*N*	96	89	85	78	75
	Mean (±SD)	59.29 (±16.95)	63.26 (±18.98)	63.06 (±18.47)	61.61 (±17.13)	58.47 (±19.89)
	Range (min, max)	15, 98	13, 104	8, 98	17, 106	3, 108
	% floor, % ceiling	0.0, 0.0	0.0, 0.0	0.0, 0.0	0.0, 0.0	0.0, 1.3
EQ-5D-5L utility (UK index) Theoretical range (min, max): −0.281, 1	*N*	96	88	85	77	74
	Mean (±SD)	0.766 (±0.19)	0.792 (±0.18)	0.790 (±0.19)	0.794 (±0.16)	0.760 (±0.22)
	Range (min, max)	−0.102, 1.000	0.103, 1.000	−0.218, 1.000	0.338, 1.000	−0.213, 1.000
	% floor, % ceiling	0.0, 10.4	0.0, 18.2	0.0, 18.8	0.0, 18.2	0.0, 17.6
EQ-5D-5L utility (NL index) Theoretical range (min, max): −0.446, 1	*N*	96	88	85	77	74
	Mean (±SD)	0.702 (±0.23)	0.736 (±0.22)	0.744(±0.23)	0.752(±0.19)	0.709(±0.25)
	Range (min, max)	−0.289, 1.000	−0.077, 1.000	−0.391, 1.000	0.195, 1.000	−0.326, 1.000
	% floor, % ceiling	0.0, 10.4	0.0, 18.2	0.0, 18.8	0.0, 18.2	0.0, 17.6

*SD* standard deviation, *FACT-G* Functional assessment of cancer therapy-general, *FACIT* functional assessment of chronic illness therapy, *FAACT* functional assessment of anorexia/cachexia treatment, *FACIT-F* functional assessment of chronic illness therapy-fatigue, *PWB* physical wellbeing, *FWB* functional wellbeing, *EWB* emotional wellbeing, *SWB* social/family wellbeing, *ACS* Anorexia-Cachexia Subscale, *TOI* trial outcome index, *EQ-5D-5L* EuroQol five-dimension five-level, *UK* United Kingdom, *NL* Netherlands

### Regression models

Table 3a, b shows parameter estimates for all mapping functions obtained from the development sample (76 patients, 332 observations) using the English and Dutch value sets, respectively. Models with squared terms showed poorer goodness-of-fit (i.e. higher QIC) compared to models without and accordingly were not retained in the analyses (results not shown). Based on QIC results, autoregressive correlation was chosen within the GEE model by assuming that repeated measures were more strongly correlated when close together in time. The preliminary analyses testing all demographic and clinical variables are not reported; among them, only ECOG performance status (score = 2 vs. score = 0–1) was included in the final models after backward selection, with higher ECOG scores predicting lower EQ-5D-5L utility values (negative coefficient; *p* < 0.001).

**Table 3:** GEE coefficient estimates (development sample)

	Model 1		Model 2		Model 3		Model 4		Model 5	
	*β*	SE	*β*	SE	*β*	SE	*β*	SE	*β*	SE
(a) UK weights										
FACT-G										
Intercept	0.5387***	0.0485	–	–	0.6185***	0.0517	**–**	**–**		
FACT-G	0.0041***	0.0007	–	–			**–**	**–**		
PWB			–	–	0.0094***	0.0019	–	–		
FWB			–	–	0.0052**	0.0019	–	–		
SWB			–	–	−0.0044*	0.0022	–	–		
EWB			–	–	0.0035	0.0022	–	–		
ECOG	−0.1865***	0.0321	–	–	−0.1586***	0.0310	**–**	**–**		
FAACT										
Intercept	0.4952***	0.0467	0.5088***	0.0474	0.5841***	0.0524	0.5210***	0.0392	0.5240***	0.0390
FAACT	0.0033***	0.0004								
FACT-G			0.0022**	0.0008						
PWB					0.0071**	0.0021			0.0077***	0.0020
FWB					0.0040*	0.0020			0.0030	0.0018
SWB					−0.0042	0.0022				
EWB					0.0021	0.0023				
ACS			0.0051***	0.0013	0.0035**	0.0013			0.0037**	0.0013
TOI							0.0046***	0.0006		
ECOG	−0.1738***	0.0310	−0.1702***	0.0308	−0.1524***	0.0304	−0.1607***	0.0305	−0.1584***	0.0304
FACIT-F										
Intercept	0.5000***	0.0433	0.5604***	0.0461	0.6157***	0.0503	0.5290***	0.0357	0.5442***	0.0363
FACIT-F	0.0033***	0.0004								
FACT-G			0.0006	0.0009						
PWB					0.0041	0.0022			0.0045*	0.0022
FWB					0.0029	0.0020			0.0017	0.0018
SWB					−0.0048*	0.0021				
EWB					0.0010	0.0022				
Fatigue			0.0074***	0.0013	0.0059***	0.0014			0.0058***	0.0013
TOI							0.0047***	0.0005		
ECOG	−0.1711***	0.0310	−0.1611***	0.0307	−0.1484***	0.0305	−0.1568***	0.0305	−0.1553***	0.0303
(b) NL weights										
FACT-G										
Intercept	0.4443***	0.0569			0.5390***	0.0609				
FACT-G	0.0049***	0.0008								
PWB					0.0107***	0.0022				
FWB					0.0066**	0.0023				
SWB					−0.0051*	0.0026				
EWB					0.0037	0.0026				
ECOG	−0.2225***	0.0385			−0.1906***	0.0377				
FAACT										
Intercept	0.3956***	0.0549	0.4098***	0.0558	0.4993***	0.0618	0.4263***	0.0462	0.4291***	0.0459
FAACT	0.0038***	0.0005								
FACT-G			0.0027**	0.0010						
PWB					0.0082**	0.0024			0.0088***	0.0023
FWB					0.0052*	0.0023			0.0040	0.0021
SWB					−0.0048	0.0025				
EWB					0.0021	0.0026				
ACS			0.0057***	0.0015	0.0039*	0.0015			0.0042**	0.0015
TOI							0.0054***	0.0007		
ECOG	−0.2078***	0.0372	−0.2040***	0.0371	−0.1834***	0.0370	−0.1925***	0.0368	−0.1901***	0.0367
FACIT-F										
Intercept	0.4019***	0.0510	0.4673***	0.0543	0.5350***	0.0593	0.4364***	0.0421	0.4520***	0.0428
FACIT-F	0.0038***	0.0005								
FACT-G			0.0010	0.0010						
PWB					0.0048	0.0026			0.0054*	0.0025
FWB					0.0040	0.0023			0.0026	0.0021
SWB					−0.0055*	0.0025				
EWB					0.0010	0.0026				
Fatigue			0.0083***	0.0015	0.0065***	0.0016			0.0064***	0.0015
TOI							0.0054***	0.0006		
ECOG	−0.2050***	0.0374	−0.1944***	0.0372	−0.1792***	0.0372	−0.1884***	0.0370	−0.1870***	0.0368

ECOG is a dummy variable assuming the value of 1 for ECOG = 2 and 0 otherwise (ECOG = 0.1)

*Model 1* algorithm using FACIT overall score(s), *Model 2* algorithm using general (FACT-G) and questionnaire-specific scale(s) separately, *Model 3* algorithm using the four FACT-G wellbeing subscales, *Model 4* algorithm using FACIT TOI score(s), *Model 5* algorithm using TOI subscales separately, *GEE* generalized estimating equations, *UK* United Kingdom, *NL* Netherlands, *SE* standard error, *FACT-G* functional assessment of cancer therapy-general, *FAACT* functional assessment of anorexia/cachexia treatment, *FACIT-F* functional assessment of chronic illness therapy-fatigue, *PWB* physical wellbeing, *FWB* functional wellbeing, *EWB* emotional wellbeing, *SWB* social/family wellbeing, ACS Anorexia-Cachexia Subscale, *TOI* trial outcome index, *ECOG* Eastern Cooperative Oncology Group

*p value <0.05

**p value <0.01

***p value <0.001

For all three FACIT instruments (FACT-G, FAACT, and FACIT-F), overall scores were highly significant (*p* < 0.001), as were the TOI scores, in models using aggregate results (Model 1 and Model 4). In models using the separate individual subscales (Model 3 and Model 5), the EWB subscale was never significantly associated with the EQ-5D-5L score; conversely, PWB had the highest explanatory value with the exception of FACIT-F Model 3, where it was not significant (*p* > 0.05). When ACS and Fatigue subscales were combined with the generic FACT-G score in models 2, the generic score was not significant in the FACIT-F model, implying that Fatigue and ECOG were sufficient to predict EQ-5D results. In all models, the coefficients of the HRQoL scales had the expected (positive) signs, indicating that better health reported by disease-specific FACT-G/FAACT/FACIT-F tools was associated with higher EQ-5D utility. The only exception was SWB that presented a negative coefficient in all models, but its value was at the limits of statistical significance.

### Model selection

A synthesis of model performances across all visits in the validation sample is reported in Table 4a, b comparing observed and predicted EQ-5D-5L utilities using English and Dutch preference weights, respectively. Overall, the mapping algorithms predicted well. The absolute differences between mean observed and mean predicted EQ-5D-5L utilities were far below the MID of 0.08 reported for EQ-5D-3L in cancer studies [26]. Moreover, none of the estimates fell outside the theoretical range of EQ-5D-5L (i.e. −0.281, 1.000) with the exception of FACIT-F Model 3 (UK), but only just (i.e. 1.014). Most of the t-test comparisons between observed and mapped scores yielded a non-significant *p* value (<0.05). However, all differences between observed and predicted values were negative, due to an overall tendency towards over-prediction in the poorest health states (EQ-5D-5L utility ≤0.700). At the same time, the range of predicted EQ-5D-5L utilities was generally narrower than the observed values and the algorithms failed to predict the value of 1 corresponding to perfect health especially when using FACT-G and FAACT, whilst considerably larger intervals were obtained through FACIT-F regression models.

**Table 4:** Synthesis of model performance across all visits (validation sample)

	Mean (±SD)	Range (min, max)	% floor, % ceiling	Mean observed − mean predicted (*p* value^a^)	MAE	RMSE
(a) UK weights						
Observed	0.809 (±0.16)	0.128, 1.000	0.0, 14.8	–	–	–
FACT-G						
Model 1	0.816 (±0.06)	0.590, 0.953	0.0, 0.0	−0.0070 (0.7017)	0.0997	0.1339
Model 3	0.832 (±0.07)	0.583, 0.970	0.0, 0.0	−0.0226 (0.2335)	0.0942	0.1272
FAACT						
Model 1	0.827 (±0.06)	0.645, 0.984	0.0, 0.0	−0.0178 (0.3393)	0.1027	0.1366
Model 2	0.825 (±0.06)	0.646, 0.971	0.0, 0.0	−0.0161 (0.3857)	0.1064	0.1412
Model 3	0.835 (±0.07)	0.638, 0.987	0.0, 0.0	−0.0259 (0.1722)	0.0995	0.1332
Model 4	0.819 (±0.07)	0.641, 0.967	0.0, 0.0	−0.0100 (0.5927)	0.1027	0.1362
Model 5	0.831 (±0.07)	0.652, 0.979	0.0, 0.0	−0.0219 (0.2455)	0.1015	0.1340
FACIT-F						
Model 1	0.831 (±0.07)	0.544, 0.998	0.0, 0.0	−0.0223 (0.2417)	0.0933	0.1267
Model 2	0.843 (±0.08)	0.575, 0.991	0.0, 0.0	−0.0343 (0.0761)	0.0942	0.1288
Model 3	0.844 (±0.08)	0.577, 1.014	0.0, 1.1	−0.0351 (0.0729)	0.0913	0.1261
Model 4	0.833 (±0.08)	0.543, 0.999	0.0, 0.0	−0.0241 (0.2161)	0.0899	0.1230
Model 5	0.842 (±0.08)	0.553, 0.998	0.0, 0.0	−0.0328 (0.0917)	0.0917	0.1257
(b) NL weights						
Observed	0.760 (±0.19)	−0.073, 1.000	0.0, 14.8	–	–	–
FACT-G						
Model 1	0.766 (±0.07)	0.505, 0.939	0.0, 0.0	−0.0161 (0.4561)	0.1094	0.1574
Model 3	0.784 (±0.08)	0.499, 0.945	0.0, 0.0	-0.0245 (0.2719)	0.1068	0.1503
FAACT						
Model 1	0.778 (±0.07)	0.568, 0.958	0.0, 0.0	−0.0178 (0.2991)	0.1131	0.1601
Model 2	0.780 (±0.07)	0.572, 0.950	0.0, 0.0	−0.0200 (0.2586)	0.1174	0.1651
Model 3	0.788 (±0.08)	0.567, 0.964	0.0, 0.0	−0.0280 (0.0932)	0.1112	0.1566
Model 4	0.776 (±0.08)	0.567, 0.950	0.0, 0.0	−0.0165 (0.3365)	0.1145	0.1605
Model 5	0.787 (±0.08)	0.576, 0.961	0.0, 0.0	−0.0266 (0.1118)	0.1126	0.1571
FACIT-F						
Model 1	0.784 (±0.08)	0.453, 0.976	0.0, 0.0	−0.0238 (0.1329)	0.1026	0.1481
Model 2	0.807 (±0.09)	0.488, 0.983	0.0, 0.0	−0.0471 (0.0030)	0.1051	0.1515
Model 3	0.797 (±0.10)	0.492, 0.992	0.0, 0.0	−0.0370 (0.0175)	0.1030	0.1471
Model 4	0.786 (±0.10)	0.453, 0.976	0.0, 0.0	−0.0262 (0.0885)	0.1007	0.1440
Model 5	0.798 (±0.09)	0.464, 0.980	0.0, 0.0	−0.0383 (0.0132)	0.1020	0.1464

*UK* United Kingdom, *NL* Netherlands, *SD* standard deviation, *FACT-G* functional assessment of cancer therapy-general, *FAACT* functional assessment of anorexia/cachexia treatment, *FACIT-F* functional assessment of chronic illness therapy-fatigue, *MAE* mean absolute error, *RMSE* root mean squared error

^a^*T* test

Best performing algorithms were identified for each FACIT questionnaire according to the lowest MAE/RMSE scores, namely Models 3 for FACT-G and FAACT, and Model 4 for FACIT-F (both for English and Dutch values). Scatterplots displaying observed and predicted EQ-5D-5L utility scores for the three best performing models applied to the validation sample (20 patients, 88 observations) are shown in Fig. 1a, b.

## Discussion

In the absence of EQ-5D or other preference-based HRQoL measurements, mapping is a useful tool in order to estimate utility values to be adopted in cost-effectiveness analyses. Over the past decade, there has been a rapid increase of mapping studies predicting generic preference-based scores from non-preference based, disease-specific results. In the area of oncology, eight studies have mapped EQ-5D from the FACIT questionnaires: three studies [11, 22, 27] mapped the general FACT-G version in various types of cancer (including lung cancer); another three algorithms [19, 21, 28] used the prostate-specific (FACT-P) module, while the remaining two adopted the melanoma-specific (FACT-M) [29] and breast-specific (FACT-B) [30] versions. Until now, no algorithms were available to map EQ-5D scores from FAACT, which is the FACIT tool specifically aimed at measuring HRQoL in patients with cancer cachexia; nor is there a mapping algorithm for the FACIT-F. Moreover, the existing algorithms map to the EQ-5D-3L rather than the EQ-5D-5L; thus, it was not possible to apply these functions to our database of EQ-5D-5L utilities. In addition, most mapping studies have used cross-sectional data and Ordinary Least Square (OLS) regression in modeling FACIT scores; even when data from multiple time points were available, only baseline information were used [21] or repeated observations were pooled together in order to increase the sample size [31].

This study, following the approach of a previous one [19], estimated GEE models to account for the longitudinal nature of the data. To increase the usability of the mapping algorithms, common demographics (e.g. age) and clinical variables (e.g. BMI), which are likely to be collected in clinical studies dealing with cancer cachexia or NSCLC were initially tested in the models. Among them, only ECOG performance score displayed a significant coefficient (*p* < 0.05) and was retained in the final models. Moreover, in addition to developing a mapping function to predict EQ-5D-5L utilities from FAACT and FACIT-F, we provided separate algorithms for the general instrument (FACT-G). Disaggregated models predicting EQ-5D-5L utility values from individual FACIT subscales were found to have better predictive ability in the case of FACT-G and FAACT, while the best performing algorithm for FACIT-F was that modeling the TOI score. These results are aligned with the current mapping literature which has shown greater explanatory power from regression models using disaggregated information instead of summary scores from a disease-specific measure [22]. Nevertheless, all the algorithms performed quite well; overall, MAE and RMSE values were comparable across the models and lower than those reported by other mapping studies [19, 24, 32]. No considerable differences were found between the algorithms developed using the English and the Dutch value sets, which identified the same best performing models (Model 3, Model 3, Model 4) within each FACIT instrument; however, mapping using English weights performed slightly better in terms of lower MAE/RMSE and smaller differences between mean observed and mean predicted EQ-5D-5L utilities. The estimated coefficients are aligned with those reported by a previous study [11] mapping FACT-G in cancer patients affected by breast, colorectal or lung cancer, where regression coefficients for the overall score were between 0.005 and 0.008 (according to the technique adopted) and coefficients for individual subscales fell in the interval 0.006–0.013 for PWB, 0.005–0.010 for FWB, 0.002–0.008 for EWB. Our slightly lower estimates may be a consequence of the different regression method (GEEs) applied, or possibly is reflecting differences in the patient groups, for example, in terms of severity.

This study presents a few limitations. First, in calculating EQ-5D-5L utilities, we adopted country preference weights that may not be the best estimates for Hungarian and Polish populations. However, due to unavailability of EQ-5D-5L tariffs for these two countries, we selected the English value set (mappings to the EQ-5D-3L have most commonly used this set) and the Dutch one, which are the two EQ-5D-5L sets of weights closest to the ROMANA trials populations [7].

Second, model validation used a non-random split-sample method, rather than testing the algorithms in an external dataset that would be the preferred approach according to the MAPS Statement [13]. However, significant differences were found in relevant baseline patients characteristics (i.e. BMI, weight loss, ECOG, chemotherapy/radiotherapy status) between the two samples, thus the cross-sample validation was likely to have been conducted on a quasi-different NSCLC-C population. A completely different approach has been suggested by most recent guidelines [33], which recommend not splitting the sample for validation purposes if this implies a further reduction of a (small) sample size.

Third, the mapping functions were developed using a small database, since only 96 patients enrolled in the ROMANA trials were invited to complete the EQ-5D-5L. However, because the patients completed the HRQoL questionnaires on up to five occasions, 420 observations were available for analysis, 79% (*n* = 332) of which were used to obtain regression coefficients and 21% (*n* = 88) to validate the mapping algorithms.

Fourth, the sample of NSCLC-C patients who were likely to have a better health status (i.e. ECOG ≤2, life expectancy at least of 4 months) than the general population with the same medical condition due to the ROMANA trials’ inclusion criteria. Thus, the generalizability of the developed mapping functions to other NSCLC-C samples should consider any potential clinical differences.

Finally, as already observed in the literature [11, 34], all mapping algorithms tend to over-predict utility values for patients in poor health and, conversely, under-predict the highest scores. Moreover, they systematically fail to predict the value of 1 corresponding to perfect health, although in our database, due to the severity of the NSCLC-C condition, less than 20% of EQ-5D-5L observations were at the ceiling at each study visit, which is lower than observed in another mapping study using the same tool [30]. This bias affected the FACIT-F algorithms less than FACT-G and FAACT ones and, within the same FACIT instrument, models using disaggregated scales (Model 3) instead of summary scores (Model 1). Skaltsa et al. [19] developed group-specific models according to disease severity to increase prediction accuracy at the “extremes”; unfortunately, due to the small database, this approach was infeasible in this study. As population-average models, the estimated GEE functions performed well in predicting mean utility values, which are usually required to populate model-based economic evaluations. New techniques such as beta-binomial regression and limited dependent variable mixture (LDVM) models [22, 23, 35], which better fit the typical distributions of EQ-5D data, are emerging in the mapping literature to overcome the well-known limitations of linear models and might be explored in future research with larger databases.

## Conclusion

Given the increasing costs of cancer care it is important to support the health-related decision-making process of allocating scarce resources by assessing the value of treatments through economic evaluation techniques such as cost-utility analysis [36]. Previous research showed that cancer cachexia, mainly affecting lung cancer patients, has a significant impact on patients HRQoL and healthcare resources utilization [10]. This study provided algorithms to predict EQ-5D-5L utility values from FACT-G, FAACT and FACIT-F scores, confirming that mapping may represent a useful tool in the absence of preference-based HRQoL scores. These algorithms could be applied in other studies related to cancer cachexia or NSCLC in general, by those requiring EQ-5D-5L utility values for QALY calculation.

## Electronic supplementary material

Below is the link to the electronic supplementary material.

Supplementary material 1 (PDF 66 kb)

Supplementary material 2 (DOCX 22 kb)

Supplementary material 3 (DOCX 18 kb)
