# Supplementary material for: Mapping health-related quality of life scores from FACT-G, FAACT, and FACIT-F onto preference-based EQ-5D-5L utilities in non-small cell lung cancer cachexia
Source: Eur J Health Econ. 2017 Sep 25;20(2):181–93. doi: 10.1007/s10198-017-0930-6 (PMC6438942; doi:10.1007/s10198-017-0930-6)

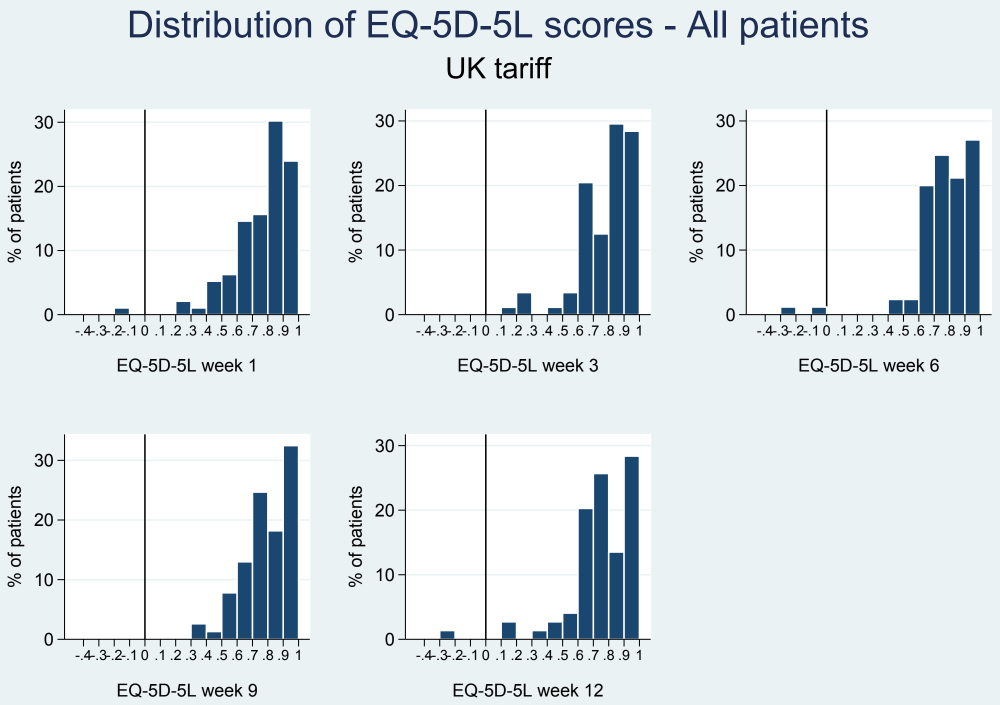

Distribution of EQ-5D-5L scores - All patients
UK tariff
% of patients
EQ-5D-5L week 1
EQ-5D-5L week 3
EQ-5D-5L week 6
EQ-5D-5L week 9
EQ-5D-5L week 12

Distribution of EQ-5D-5L scores - All patients

NL tariff

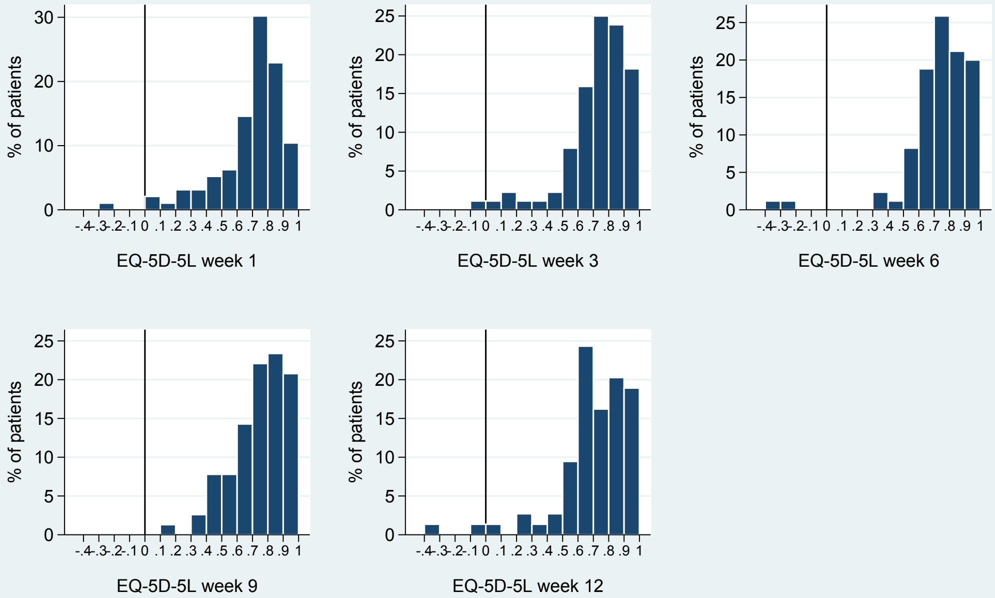

Supplement: Supplementary file 1 — Supplementary material 1 (PDF 66 kb) [file 10198_2017_930_MOESM1_ESM.pdf]
