# Supplementary material for: Mapping health-related quality of life scores from FACT-G, FAACT, and FACIT-F onto preference-based EQ-5D-5L utilities in non-small cell lung cancer cachexia
Source: Eur J Health Econ. 2017 Sep 25;20(2):181–93. doi: 10.1007/s10198-017-0930-6 (PMC6438942; doi:10.1007/s10198-017-0930-6)
Supplement: Supplementary file 3 — Supplementary material 3 (DOCX 18 kb) [file 10198_2017_930_MOESM3_ESM.docx]

**Table A2.** Pearson correlation coefficients between FACIT scores (source measure) and EQ-5D-5L utilities (target measure) by study visits (overall sample).

|  | **Baseline** | | **Week 3** | | **Week 6** | | **Week 9** | | **Week 12** | |
| --- | --- | --- | --- | --- | --- | --- | --- | --- | --- | --- |
| **Variables** | **EQ-5D-5L utility (UK index)** | **EQ-5D-5L utility (NL index)** | **EQ-5D-5L utility (UK index)** | **EQ-5D-5L utility (NL index)** | **EQ-5D-5L utility (UK index)** | **EQ-5D-5L utility (NL index)** | **EQ-5D-5L utility (UK index)** | **EQ-5D-5L utility (NL index)** | **EQ-5D-5L utility (UK index)** | **EQ-5D-5L utility (NL index)** |
| **FACT-G** | 0.4780* | 0.4864* | 0.4301* | 0.4341* | 0.5506* | 0.5599* | 0.3733* | 0.3761* | 0.5711* | 0.5649* |
| **FAACT (overall)** | 0.5383* | 0.5465* | 0.4731* | 0.4780* | 0.6023* | 0.6057* | 0.4057* | 0.4067* | 0.5839* | 0.5751* |
| **FAACT (ACS)** | 0.5019* | 0.5078* | 0.4365* | 0.4420* | 0.5957* | 0.5891* | 0.3776* | 0.3758* | 0.4780* | 0.4666* |
| **FAACT (TOI)** | 0.5442* | 0.5491* | 0.5122* | 0.5171* | 0.6103* | 0.6100* | 0.4739* | 0.4736* | 0.5946* | 0.5819* |
| **FACIT-F (overall)** | 0.5671* | 0.5720* | 0.5177* | 0.5202* | 0.6135* | 0.6204* | 0.4447* | 0.4381* | 0.6005* | 0.5957* |
| **FACIT-F (Fatigue)** | 0.6097* | 0.6087* | 0.5583* | 0.5580* | 0.6016* | 0.6042* | 0.4586* | 0.4404* | 0.5952* | 0.5911* |
| **FACIT-F (TOI)** | 0.5948* | 0.5957* | 0.5660* | 0.5677* | 0.6244* | 0.6288* | 0.5068* | 0.4968* | 0.6232* | 0.6156* |
| **PWB** | 0.4909* | 0.5020* | 0.5494* | 0.5514* | 0.5600* | 0.5613* | 0.4558* | 0.4500* | 0.5541* | 0.5434* |
| **SWB** | 0.2105* | 0.2296* | 0.1062 | 0.1208 | 0.0687 | 0.0859 | -0.0653 | -0.0519 | 0.166 | 0.1731 |
| **EWB** | 0.3270* | 0.3279* | 0.3134* | 0.3053* | 0.5080* | 0.5088* | 0.3150* | 0.3074* | 0.4917* | 0.4890* |
| **FWB** | 0.3037* | 0.2977* | 0.2741* | 0.2798* | 0.3519* | 0.3604* | 0.3471* | 0.3562* | 0.4978* | 0.4868* |

*p-value <0.05

*Abbreviations*: FACT-G: Functional Assessment of Cancer Therapy – General; FAACT: Functional Assessment of Anorexia/Cachexia Treatment; FACIT-F: Functional Assessment of Chronic Illness Therapy-Fatigue; PWB: physical wellbeing; FWB: functional wellbeing; EWB: emotional wellbeing; SWB: social/family wellbeing; ACS: Anorexia-Cachexia Subscale; TOI: trial outcome index; EQ-5D-5L: EuroQol five-dimension five-level; UK: United Kingdom; NL: Netherlands.
